# Toward a Model for Measuring Social and Structural Determinants of Alzheimer’s Disease and Related Dementias

**DOI:** 10.1093/geroni/igab046.303

**Published:** 2021-12-17

**Authors:** Shana Stites, Joyce Balls-Berry, Lisa Barnes

## Abstract

Social and structural determinants of health (SSDoH) are conditions in the environments in which individuals are born, live, learn, work, play, worship, and age that affect health, functioning, and quality-of-life outcomes across the life course. Growing evidence suggests that SSDoH can help to explain heterogeneity in cognitive, functional, and interventional outcomes in Alzheimer’s disease and related disorders research and clinical practice. The National Institute on Aging (NIA) has prioritized collecting SSDoH data in order to elucidate disease mechanisms and aid discovery of a disease modifying treatments. However, a major nexus of ADRD research, the national network of Alzheimer’s Disease Research Centers (ADRCs), does not routinely collect SSDoH data. We describe a model for feasibly gathering and analyzing SSDoH data across Alzheimer’s Disease Research Centers (ADRCs). We lay out theoretical underpinnings of key constructs and their measure, empirical evidence for their importance, and their potential for elucidating disease and prevention mechanisms. Toward a goal of translation, we describe a general approach to measuring SSDoH along with core set of measures. We also describe empirical support and rationales for assessing SSDoH in standing geographically and culturally diverse research cohorts, and guiding considerations in selecting modules to serve unique communities. We specifically address SSDoH in Black, Hispanic/Latin, and refugee populations with an eye toward conveying how geographic proximity, socioeconomic status, ethnoracial factors, and sex\gender\sexual orientation affect populations in ways directly relevant to Alzheimer’s disease (AD) and Alzheimer’s disease related dementias (ADRD).

